# Application of an Online-Biomass Sensor in an Optical Multisensory Platform Prototype for Growth Monitoring of Biotechnical Relevant Microorganism and Cell Lines in Single-Use Shake Flasks

**DOI:** 10.3390/s140917390

**Published:** 2014-09-17

**Authors:** Christian Ude, Jörg Schmidt-Hager, Michael Findeis, Gernot Thomas John, Thomas Scheper, Sascha Beutel

**Affiliations:** 1 Leibniz University of Hanover, Institute of Technical Chemistry, Callinstr. 5, 30167 Hannover, Germany; E-Mails: ude@iftc.uni-hannover.de (C.U.); Schmidt-hager@iftc.uni-hannover.de (J.S.-H.), scheper@iftc.uni-hannover.de (T.S.); 2 PreSens Precision Sensing GmbH, Josef-Engert-Str. 11, 93053 Regensburg, Germany; E-Mails: Michael.Findeis@presens.de (M.F.); G.John@presens.de (G.T.J.)

**Keywords:** biomass sensor, light scattering, optical density, process monitoring, shake flask

## Abstract

In the context of this work we evaluated a multisensory, noninvasive prototype platform for shake flask cultivations by monitoring three basic parameters (pH, pO_2_ and biomass). The focus lies on the evaluation of the biomass sensor based on backward light scattering. The application spectrum was expanded to four new organisms in addition to *E. coli K12* and *S. cerevisiae* [1]. It could be shown that the sensor is appropriate for a wide range of standard microorganisms, e.g., *L. zeae, K. pastoris, A. niger* and CHO-K1. The biomass sensor signal could successfully be correlated and calibrated with well-known measurement methods like OD_600,_ cell dry weight (CDW) and cell concentration. Logarithmic and Bleasdale-Nelder derived functions were adequate for data fitting. Measurements at low cell concentrations proved to be critical in terms of a high signal to noise ratio, but the integration of a custom made light shade in the shake flask improved these measurements significantly. This sensor based measurement method has a high potential to initiate a new generation of online bioprocess monitoring. Metabolic studies will particularly benefit from the multisensory data acquisition. The sensor is already used in labscale experiments for shake flask cultivations.

## 1. Introduction

### 1.1. Online Process Monitoring in Shake Flasks

Shake flasks are still used in biotechnology because of their easy handling, low cost and simple setup. The most common process measurements, all of which are applied by sampling and offline-measurements for shake flask growth monitoring, are: Optical density, CDW (cell dry weight) and cell concentration. In the past there has been little research on online bioprocess monitoring for shake flasks. Extensive analytics within the flask represented rather too much equipment considering the original simple concept of shake flasks. Electrochemical electrodes used for bioreactors are not suitable due to their size and their influence on the liquid-dynamics. That causes major problems in terms of full immersion of the electrodes or other submersed sensors. The idea of a minimum space demand and totally non-invasive measuring system led to the development of optical chemosensors in combination with optical fibers.

### 1.2. Optical Biomass Monitoring Methods and Devices

Online biomass monitoring via scattered light has several advantages over offline sampling. Apart from the general advantages of noninvasive, nondestructive online monitoring there are special advantages concerning the combination of light and shake flasks. Especially in small scale systems, for instance widely used shake flasks, sampling volume is highly limited while opening and removal of liquid represents a significant disturbance of the whole process.

The first *in situ* online biomass sensors were developed for large fermenters. Different types of optical sensor probes are commonly used in bioreactors [2–4]. The main differences concern wavelength, the geometric arrangement and the angle between light source and sensor. The angle determines the sensitivity and specificity of the measured signal. Turbidity probes measure the absorbance (like photometers) with a constant light path at a 0° angle. They exhibit a small linear range because of the Beer-Lambert law. Measuring scattered light instead of absorbance offers a wider linear range compared to turbidity. In general the measurement using an 90° arrangement (nephelometry) is more sensitive at low cell concentrations, while backward scattered light (often stated as 160°–180° scattered light) still exhibits a linear correlation at high cell concentrations [2,3]. Apart from sensitivity, the specificity for certain parts of cells is affected by the arrangement of light source and sensor. The 90° scattered light is rather insensitive to particle size [5]. At angles >90° (towards backward scattered light) the scattered light becomes more sensitive to the nucleus and organelle concentration [6,7]. It is also more sensitive to the cell membrane structure [7]. Some devices combine turbidity- and nephelometric-measurement in one sensor [8]. When optical sensors are used, interferences are caused by bubbles, foam, biofilm formation on optical components, solids and cell aggregations [4,9,10]. While foam can be prevented using anti-foam agent, bigger bubbles can only be removed in combination with a degasser or a bypass arrangement of the sensor is needed [3]. This method is inappropriate for small scale cultivation systems and advanced data filtering is necessary to remove erroneous data points.

Biomass measurement within shake flasks is facing similar challenges compared to pH and DO-measurement. There are attempts to measure optical density in shake flasks using prisms and optical fibers *in situ* [11]. A critical limitation of this concept is the reproducible installation of the optical components and the installation of the flask on the sensor. Additionally, the components in the flask act as a disturbance of the fluid dynamics and may influence the experiment. An appropriate non-invasive measurement method can be carried out by measuring scattered light. A light cone is emitted by a LED while scattered light will be detected by a photodiode (PD) using light of 600 nm wavelength or NIR (near infrared) light range [4,9]. NIR light has the advantage of being less absorbed by soluble media components [10]. LED and PD can be arranged around the flask with a certain angle offering different sensitivities and calibration ranges. In the case of shake flasks, angles α ≤ 90° (between LED and PD) are preferable due to technical reasons. A US Patent (US 7339671) titled “Apparatus and method for monitoring biological cell culture” (published in 2005) is an example of a device which could measure the biomass concentration online [12]. It is intended to have two light sources featuring a wavelength at 650 nm for bacteria and 780 nm for mammalian cells. The angle between light source and photodiode is approx. 60° in order to enable a higher sensitivity at low cell concentrations. Another approach is the measurement using a non-invasive handheld device (Buglab “OD Scanner”). It works with near-infrared light [13]. A minimum liquid height of 3 cm is needed to sustain stable measurements. A linear calibration of CDW *versus* the biomass sensor signal from 0.05 up to 10 g·L^−1^ with an error of <15% was reportedly achieved using *E. coli* (Buglab, Concord, USA). The main disadvantages of handheld systems are the need for instructed staff (reproducibility), the lack of online measurement capability and a time consuming analysis procedure in case of parallel cultivations. Furthermore, stopping the cultivation in order to perform the measurement causes disturbance. This article investigates the applicability of a platform-embedded 180° scattered light sensor prototype (*PreSens* GmbH, Regensburg, Germany) that faces the aforementioned challenges and may be able to overcome the limitations of existing systems. Several model microorganisms and cell lines representing aerobic and facultative anaerobic bacteria, fungi, yeasts and mammalian cells were chosen for the evaluation of the sensor. The technical design and the functional principle using an acceleration sensor have been described previously [1].

## 2. Experimental Section

### 2.1. Cultivation Methods

The used microorganisms, cell lines and the applied media are listed in Table 1. All media were prepared with deionized water obtained by Arium^®^ 661 Ultrapure water system (*Sartorius Stedim Biotech* S. A, Göttingen, Germany). Media preparation [g·L^−1^]: MRS (de Man, Rogorosa and Sharpe) for *Lactobacillus* (5.0 yeast extract (AppliChem), 10.0 bacto-tryptone (BD), 20.0 glucose (Sigma-Aldrich), 5.0 natriumacetate (Fluka), 2.0 K_2_HPO_4_ (Fluka), 0.2 MgSO_4_·7H_2_O (Fluka), 0.05 MnSO_4_·7H_2_O (Merck), 1.58 citric acid (Riedel de Haën), 5.0 mL·L^−1^ vitamin solution, pH 6.5). YM (yeast and mold) (3.0 yeast extract, 5.0 malt extract (Sigma-Aldrich), 5.0 soy broth (Sigma-Aldrich), 10.0 glucose, pH 6.0). FM (fungi medium) for *Aspergillus* (10.0 soy broth, 20.0 glucose, 1.0 NaH_2_PO_4_ (Riedel de Haën), 1.0 KH_2_PO_4_ (Fluka), 5.0 NH_4_SO_4_ (Roth), 2.0 MgSO_4_·7H_2_O pH 6.0). TC42-medium (TeutoCell) for CHO-K1. 200 μl·L^−1^ of TEGO^®^ Antifoam KS 911 were added to each medium.

Vitamin-solution [mg·L^−1^]: 2.0 Biotin, 2.0 folic acid, 10.0 pyridoxine-HCl, 5.0 thiamin-HCl, 5.0 riboflavin, 5.0 nicotinic acid, 5.0 D-Ca-pantothenate, vitamin B12, 5.0 p-aminobenzoic acid, 5.0 lipoic acid.

The cultivation parameters are listed in Table 2. Standard X-ray sterilized, disposable shake flasks (Corning^®^) equipped with DO/pH-sensorspots, with baffles (WB) or without baffles (NB) were used (*PreSens* GmbH, Regensburg, Germany). Shaking was performed on an orbital shaker with 20 mm shaking radius (Certomat^®^ SII, *Sartorius Stedim Biotech* S. A, Göttingen, Germany). For mammalian cell cultivation, a shaker with 10 mm shaking radius was used (DOS-10M, *Elmi* Ltd., Riga, Latvia).

#### 2.1.1. *A. Niger* Cultivation Method

*A. niger* was grown from spores which were conserved in cryovials containing 0.9% (w/v) NaCl-solution and 5% (w/v) glycerol. Precultures were inoculated with 5 × 10^5^ spores·mL^−1^. After 20 h the mycelium balls were homogenized under sterile conditions by an ultra-turrax^®^ T25 for 2 min. For the main culture 10% (v/v) of the homogenate was used. For each CDW data point one flask was prepared and cultivated in parallel.

#### 2.1.2. CHO-K1 Cultivation Method

**Figure 1.**
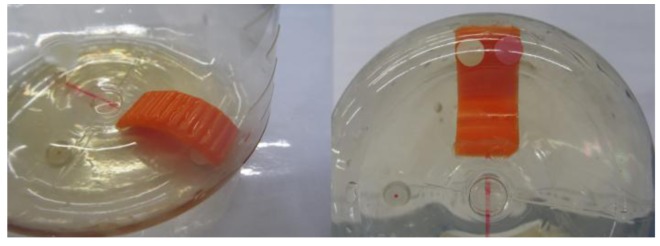
Positioning of the light shade on the bottom of a corning non-baffled 500 mL disposable shake flask. White dot = pH sensorspot, reddish dot = oxygen sensorspot.

CHO-K1 cells were grown from cryovials containing 10% (v/v) DMSO (dimethyl sulfoxide). Main cultures were inoculated with 6–7 × 10^5^ cells·mL^−1^. The cells were grown in two passages in order to adapt them to the media and cultivation conditions. In this stage cells were grown at least to 1 × 10^7^ cells·mL^−1^ in about 6 days on the sensor-platform. For passage cells with a viability of 95% − 100% were used. The growth of CHO-K1 could not be measured by the standard sensor setup because of their growth at comparably low cell densities and low optical density compared to bacteria or fungi. To avoid the crucial problem of high signal noise caused by boundary layer reflections, a small light shade was mounted into the flask (Figure 1). It was made from biocompatible high density polyethylene and has the shape of a ribbed roof. It provides a constant measuring zone and absorbs reflections from the boundary layer.

### 2.2. Offline Sampling Methods

#### 2.2.1. OD_600_ Measurement

OD measurement was performed by taking an aliquot from the culture vessel under sterile conditions. Dilutions were created with 0.9% (w/v) NaCl solution in a polystyrene cuvette (path length = 1 cm). Three dilutions were prepared and measured at 600 nm (Uvikon 922, *Kontron Instruments GmbH*, Düsseldorf, Germany). The dilution proportion was calculated under the condition that absorbance is below 0.7. The obtained values were averaged.

#### 2.2.2. CDW Determination

For CDW determination a 2 mL aliquot was taken from the culture vessel under sterile conditions and transferred into a dried, preweighted 2 mL reaction tube. The cells were centrifuged at 16,060 xg for 5 min (yeast), 6 min (*L. zeae*). The pellet was washed once with 0.9% (w/v) NaCl-Solution and centrifuged again (see above). The clean pellet was dried at 85 °C until a constant mass was measured. In case of *A. niger* the complete culture in the shake flask was harvested. The mycel was separated from the culture broth by filtration trough a dried, preweighted 20 μm pore filter sheet (Calbiochem^®^ Miracloth). The mycel was purged with autoclaved deionized water. The clean mycel was dried at 85 °C until a constant mass was measured. The CDW was calculated on base of the mass difference. The reliability of the CDW determination method for bacteria and yeast was exemplarily tested with *S. cerevisiae* NCYC 1024. A 10-fold determination with a mean biomass concentration of 5.5 g·L^−1^ was performed and standard deviation determined.

#### 2.2.3. Cell Counting

For cell counting a Thoma chamber with 20 μm depth was used. Dilutions were prepared with 0.9% (w/v) NaCl-Solution. Four 25 μm squares were counted and the cell count was multiplicated with a chamber factor of 2 × 10^5^ in order to obtain the cell concentration in cells per mL. Dilutions were prepared in a way that cell count should not exceed 100 cells per 4 big squares. In the case of *L. zeae* cells were counted manually. Cells of *K. pastoris* were counted by taking two pictures of the loaded counting chamber per sample with a CCD-camera. Cells on the pictures were counted according to Section 2.2.4. CHO-K1 cells were counted automatically with the cell counter AS20 (Cedex Bio HT, *Hoffmann-La Roche*, Basel, Switzerland). Cell solutions were automatically mixed with trypan blue.

#### 2.2.4. Cell Size Determination

During cultivation pictures of 1280 × 960 pixels were taken of a loaded Thoma chamber (1 pixel ≙ 0.532 μm). Therefore the whole frame is equivalent with 347,669 μm^2^. The chamber factor was calculated with 143,815. Digital image processing was performed by the software *ImageJ.* That included the application of standard operations: Edge detection by Laplace-operator, binarization, opening, Watershed-algorithm (see supplementary material and data deposit). For each organism a picture of two independent cultivations was chosen. The pictures were taken during exponential growth phase. The determined area of all cells within the picture was divided by their detected count. The averaged cell plain was exponentiated by 0.5 in order to get an averaged cell size.

### 2.3. Technical Sensor Specifications

The embedded LED model used was a NSPR310S (*Nichia* Corp.), the photodiode was a GSI10530 (*General Semiconductor Industries* Ltd), the optical filter was a Calflex^TM^ C, the cover glass was a 1 mm Schott OG590. The distance between LED and photodiode was 7.2 mm.

### 2.4. Parameters of Online Biomass Monitoring Using the SFR

The presented experiments were performed with two types of disposable shake flasks (Corning) including sensorspots as follows:

(1) SFS-HP5-PSt3-500-WB-VEC-v3 ID 11-34-01.
(2) SFS-HP5-PSt3-500-NB-VEC-v3 ID 11-07-04.

The default sensorspot calibrations were:

(1) Phase 0% air sat [°] 58.04, Phase 100 [°] 24.40, Temp 0 [°C] 36.70, Temp 100 [°C] 36.70, P [mbar] 975.00, pHmax 22.27, pHmin 55.03, pHTemp 37.00, dpH 0.56, pH0 6.67.
(2) Phase 0% air sat [°] 55.16, Phase 100 [°] 24.17, Temp 0 [°C] 37.10 Temp 100 [°C] 37.10, P [mbar] 956.00, pHmax 22.06, pHmin 55.16, pHTemp 37.1, dpH 0.55, pH0 6.66.

The trigger angle configurations were: –134 (*L. zeae*), 0 (*K. pastoris, A. niger*), –66 (CHO-K1). The measuring interval was 7 s while amplitude correction was set to 1.0 (bacteria, yeast, fungi), 59 s and 0.01 (CHO-K1).

### 2.5. Dynamic Calibration Procedure

Dynamic calibration was performed by monitoring cultivation with classical offline methods and online measurement at the same time. The collected data were calibrated subsequently against each other. Sampling was generally carried out in intervals of one hour. In the case of CHO-K1 and *A. niger* twice a day. Due to the high amount of online data a set of individual VBA macros were used for calibration. The aim was up to calibrate the biomass sensor signal against CDW, OD_600_ and cell concentration. The biomass sensor signal was forward-median-filtered with a frame of 45 data points directly after the sampling procedure. The applied calibration algorithm to find the correspondent offline value is a “vlookup” similar searching algorithm (see Supplementary Material and Data Deposit). In the case of *A. niger* one flask for each offline biomass measurement was prepared and parallel cultivations were carried out. The flasks were placed on the sensor and at least 45 data points for each calibration point were recorded. No lag-phase data was included in the calibration process. Cultivations were independently performed three times. The data of two cultivations were used for data fitting, while the data of the third cultivation was used for validation. In the case of CHO-K1 one cultivation was used for calibration. The data fitting was achieved by minimization of the χ^2^ value by the Levenberg-Marquardt-Algorithm (using *Origin*^®^ *8.5*, OriginLab). Three types of functions proved to be adequate (Equations (1)–(3)).

(1) $$f\left(x,a,b,c\right)={\left(a+bx\right)}^{-\frac{1}{c}}$$

(2) f(x,a,b,c)=a−b⋅ln(x+c)

(3) $$f\left(x,\;a,b,c,d,e\right)=a+bx+cx^{2}+dx^{3}+ex^{4}$$

### 2.6. Validation Method of Dynamic Calibrations

The calibrations were validated by their reproducibility and their root mean square error of prediction (RMSEP) (Equation (4)). In this case m_i_ represents the offline data which was part of the calibration (see Section 2.2), *f* is the fitting function of the plot: “Offline measurement *versus* biomass sensor signal”. *p_j_* represents the different parameters (a, b, c …) which are part of the specific fitting functions. The percentaged error was calculated according to Equation (5).

(4) $$\text{RMSEP}=\;\sqrt{\frac{\sum_{i=1}^{n}{\left(f\left(x_{i},\;p_{j}\right)-m_{i\;}\right)}^{2}}{n}}$$

(5) $$\text{rror}\;\left[\%\right]=\;\frac{\sum_{i=1}^{n}\frac{\left|f\left(x_{i},p_{j}\right)-m_{i\;}\right|}{f\left(x_{i},p_{j}\right)}}{n}\cdot 100$$

### 2.7. Static Calibration Procedure

The static calibration method was used to determine the upper threshold of the biomass sensor. Biomass was produced by cultivations in complex media at early stationary growth phase at OD_600_ = 26. The cell suspension was centrifuged at 2000 xg for 10 min (yeast) in 50 mL reaction tubes. The media was discarded, while the cell pellet was washed once in 0.9% (w/v) NaCl-solution. 10 mL of saline were added to the pellet and shortly, vigorously mixed. The milky supernatant was discarded. This procedure was repeated twice. The pellet was used to prepare sequential dilutions which were measured with the sensor, collecting 45 data points.

## 3. Results and Discussion

### 3.1. Application on Different Cell Strains

#### 3.1.1. Calibration Characteristics and Data Fitting

**Figure 2.**
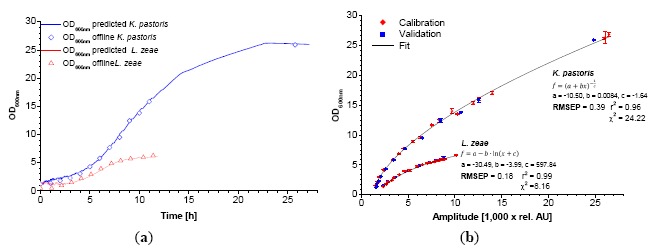
Exemplary dynamic calibration of the biomass sensor signal *versus* optical density at λ = 600 nm for *K. pastoris* and *L. zeae* (**a**). Red diamonds represent two experiments. Blue squares represent one validation experiment. Application of the calibration function on the biomass sensor signal of the validation growth curve along with the offline measured data (**b**).

The used scattered light sensor proved its applicability to monitor the growth of different model organisms and cell lines commonly used in biotechnology. As previously described, the calibration against conventional methods of cultivation measurement has been successful for *E. coli* K12 and yeast [1]. In this new report, the application range was extended to *K. pastoris, L. zeae, A. niger* and CHO-K1 cells. As shown in Figure 2, calibration of the biomass sensor signal *versus* OD_600_ exhibits a significant difference between the data sets of *K. pastoris and L. zeae.* The latter holds an approx. two times higher biomass sensor signal ratio compared to the corresponding OD_600_ values. This difference increases towards higher OD_600_ values.

**Figure 3.**
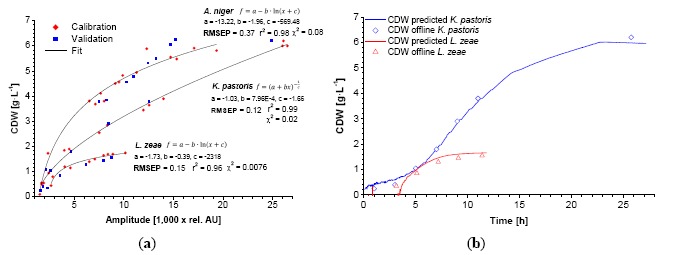
Exemplary dynamic calibration of the biomass sensor signal *versus* CDW for *K. pastoris, A. niger* and *L. zeae* (**a**). Red diamonds represent two experiments. Blue squares represent one validation experiment. Application of the calibration function on the biomass sensor signal of the validation growth curve along with the offline measured data (**b**).

The characteristics of the calibration functions used for data fitting are similar for both cases. In the case of *K. pastoris*, the 3-parametric power function named after Bleasdale and Nelder (Equation (1)) results in the lowest χ^2^ [14]. The calibration of *L. zeae* could be better described with a 3-parametric logarithmic function (Equation (2)). A similar correlation was found during calibration of the CDW (Figure 3). Smaller stick-like cells (*L. zeae* 2.2 μm) exhibit more scattered light than round yeast cells (*K. pastoris* 3.7 μm) per OD_600_ and CDW. The mycelium of *A. niger* showed the lowest scattered light per biomass.

**Figure 4.**
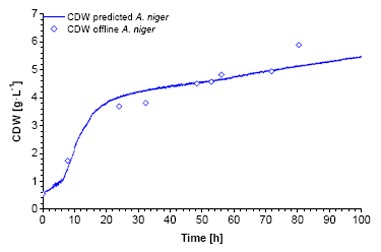
Application of the calibration function of *A. niger* on the biomass sensor signal of the validation growth curve along with the offline measured data.

The bended character of the calibrations is explained by the nature of 180° scattered light measurements in a vessel. It is known that OD_600_-CDW correlations can be generally described with linear functions for homogenous dilutions of cells. In contrast, the sensor light cone, generated by the LED, has a limited invasion depth into the suspension which follows the Lambert-Beer's-law. At very low biomass concentrations (approx. < 0.4 g·L^−1^) the light invades deep into the suspension and beyond, causing signal noise due to boundary layer reflections. These data points were excluded from the calibration. At low biomass concentrations the scattered light is induced by the entire light cone. The cell concentration close to the photodiode is comparatively low. In this case the light has to travel a relative long way to the sensor, comprising a loss in intensity (absorption). Towards higher concentrations the invasion depth decreases. The majority of the light is scattered by the cells which are close to the vessel wall, *i.e.*, close to the photodiode. The biomass sensor signal is subsequently increasing faster at higher biomass concentrations. In the case of *K. pastoris,* the bended character of the calibration maintains until 6 g·L^−1^. The application of the created calibrations using fitting functions showed a good correlation between predicted and offline data (Figure 2b, Figure 3b, Figure 4).

#### 3.1.2. Influence of Media Composition and Sensor Detection Range

**Figure 5.**
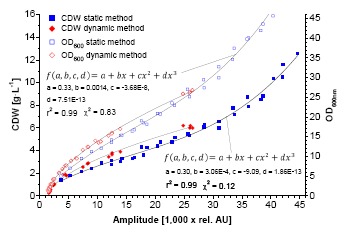
Calibration of the biomass sensor signal *versus* cell dry weight (CDW), OD_600_ using medium free cell suspension dilutions. Parameters: 500 mL WB, 100 mL vol., 150 rpm (r = 2 cm).

There also is a dependency of the used medium. In complex media, light absorption can be associated to different concentrations of yeast extract and its components respectively. Vitamins as well as Maillard-reaction byproducts formed during fabrication process and autoclaving play a major role (data not shown). In these investigations, MRS had the highest capability to reduce the scattered light by absorption. In addition, the optical properties of the medium are changing during cultivation; e.g., substances are assimilated into the cell, which affects calibrations as well. *K. pastoris* is widely used for yield biomass cultivations. In order to investigate the upper detection limits of the sensor, cells were separated from the medium, concentrated and measured in sequential dilutions with 0.9% (w/v)-NaCl-solution. A calibration up to 12.55 g·L^−1^ was possible (Figure 5) using a cubic fitting function instead of Bleasdale-Nelder. A saturation effect of the emitted scattered light above 6 g·L^−1^ could be seen. In comparison the reflectance signal of a 180° scattered light sensor probe using NIR (810 nm) showed a linear correlation up to 30 g·L^−1^ [15].

#### 3.1.3. Sensor Sensitivity in Consideration of Cell Morphology

**Figure 6.**
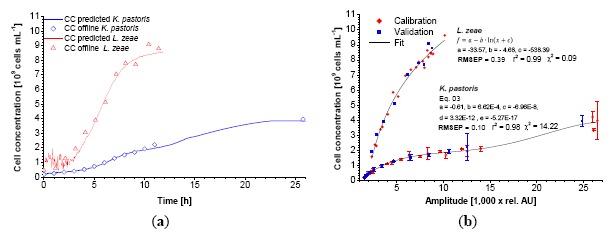
Exemplary dynamic calibration of the biomass sensor signal *versus* cell concentration for *K. pastoris* and *L. zeae*. Red diamonds represent two experiments. Blue squares represent one validation experiment (**a**). Application of the calibration function on the biomass sensor signal of the validation growth curve along with the offline measured data (**b**).

Calibration of the biomass sensor signal against the cell concentration gives a clear view of the high influence of cell morphology on the scattered light intensity. Between 1.0 and 2.0 × 10^9^ cells·mL^−1^ the generated signal of *K. pastoris* is 10-fold higher at the same cell concentration than for *Lactobacillus* (Figure 6).

Since there is little difference of the refractive index of yeast cells (1.36–1.40 [16]) compared to *Bacillus* (1.45 [17]), this correlation is more likely determined by the shape and size of the cells. The assumption of a dependency of backward scattered light on cell morphology is supported by the drift in the calibration of *K. pastoris*. A polynomial function is needed to fit the data (Equation (3)) while in the case of *L. zeae* the same fitting function as in OD_600_ and CDW-calibration can be used. The analysis of the microscopical pictures revealed that the size of *K. pastoris* was significantly decreasing because of nutrient deficiency in the stationary phase of the cultivation. This behavior was not found during cultivation of *S. cerevisiae,* while calibration against the cell concentration did not show a polynomial trend either (data not shown). For microbial organisms, CDW seems to be the most universal calibration reference because of its independence from any additional optical influences and photometer specifications. In general, OD_600_ and CDW can be used alongside because of the linearity of their correlation. Calibration against the cell concentration is only useful when cells are uniform and do not change their morphology or specific cell mass. Morphological changes would influence the backscattered light while not being visible in the cell concentration data.

### 3.2. Cultivation at Low Optical Densities

**Figure 7.**
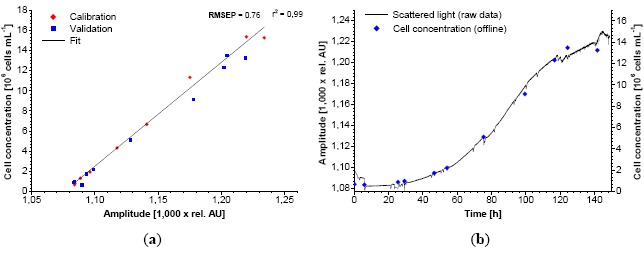
Calibration of the biomass sensor signal *versus* cell concentration using Chinese hamster ovary (CHO)-K1 and a special light shade. Red diamonds represent two experiments. Blue squares represent 1 validation experiment (**a**). Comparison between the biomass sensor signal and offline cell counting (Cedex) during the growth of CHO-K1 (**b**).

The monitoring of CHO-K1 cells with the standard sensor setup did not supply suitable calibrations in terms of a good sensitivity and dynamic range. The use of a modified shake flask (Figure 1) allowed a linear calibration against the cell concentration (Figure 7).

**Figure 8.**
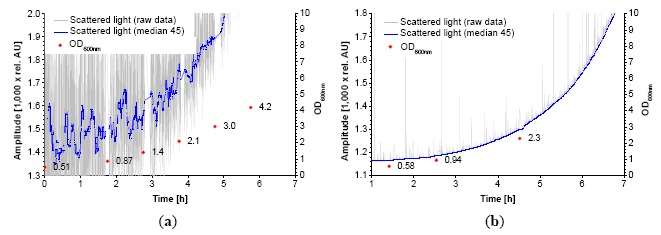
Biomass sensor signal trend during lag-phase and early exponential growth phase of *K. pastoris* with an unmodified shake flask (**a**) Same cultivation using a custom made light shade within the shake flask (**b**).

The calibration range is 0.67 × 10^6^–1.5 × 10^7^ cells·mL^−1^ which is sufficient for a complete cultivation monitoring. Lower cell concentrations could not be measured because the amount of backscattered light of the cells at 600 nm was too low. At these concentrations, the light reflected by the light shade dominates the base signal. It is likely that a concentration of >1.5 × 10^7^ cells·mL^−1^ could be measured and calibrated with this setup. NIR turbidity probes, which are common for bioreactors (aquasant AF 44, SIR Ingold FSC 402), also exhibit a linear calibration against the cell concentration [18]. At the end of the cultivation shown signal noise was apparently increasing. This effect is due to accumulation of cell debris and released organelle which also affects scattered light [19]. A light shade modification in the flask was also tested during the lag-phase of microbial cultures. As seen in Figure 8, the signal to noise ratio during the lag-phase massively increased. That enabled measurements down to OD_600_ = 0.58 (0.1 g·L^−1^ CDW). A critical parameter in this concept is the positioning of the light shade. Like the shake flask itself it has to be fabricated and fixed precisely. That could be a major drawback concerning mass production.

### 3.3. Reproducibility and Quality of Prediction

The statistical analysis of the calibrations showed that the scattered light sensor had a two to three times higher RMSEP (Table 3) compared to the standard deviation of the offline OD_600_ measurement (Table 4). Concerning CDW the RMSEP was comparable to the standard deviation. In the case of calibration against the cell concentration the RMSEP was two times higher for *L. zeae*, while it was lower for *K. pastoris.* The most precise calibrations were obtained against OD_600_ and cell concentration. The higher relative error of CDW-calibrations is rather based on the high error of the CDW-determination method at low biomass concentrations. However, CDW-calibrations should be preferred to OD_600_. CDW is more universal while OD_600_ measurement strongly depends on the used device. The range to observe is also important for the precision of the received data. Below OD_600_ = 2 (about 0.35 g·L^−1^ *K. pastoris*-CDW and 0.6 g·L^−1^ *L. zeae-*CDW with the given photometer) measurements had a low precision due to the low signal to noise ratio (Figure 8). In contrast relative errors were significantly lower at higher biomass concentrations (Table 3). Compared to bacteria and yeast, *A. niger* and CHO-K1 showed the highest RMSEP in CDW and cell concentration, respectively. This indicates that inhomogeneity of the mycel and measuring at low OD_600_ is a challenge for further developments of the sensor system. The choice of calibration (CDW, OD_600_, CC) is up to the preferences of the user. In some cases (e.g., CHO-K1) it does not make sense to use CDW or OD_600_ as reference because it is rarely used for growth monitoring. In the case of *A. niger* calibration against OD or CC does not make sense.

## 4. Conclusions/Outlook

The investigated scattered light sensor proved to be applicable for a wide range of different microorganisms and cell lines commonly used in biotechnology. The biomass sensor signal depends on the morphology of cells and the media composition used, what makes individual calibration necessary. Calibrations against CDW or cell concentration are more universal than against other optical measurement methods. Assuming that a maximum measurement error of <10% is acceptable, the sensor is valid for all examined organisms. By introduction of a simple light shade into the shake flask it was possible to measure at low optical densities in exemplary the lag-phase of microbial culture and CHO-K1 culture overall. The dependency on the cell morphology is no disadvantage; it rather provides additional information about sudden aberrations in terms of culture conditions. The advance of combining two sensors (scattered light and dissolved oxygen sensor) proved to be very effective for the observation of short-term metabolic changes which affect growth and oxygen demand as well. These features pioneer real-time pH and substrate process control in shake flasks. Thus, scattered light measurement for shake flask cultivation does not simply represent an “online” replacement for OD_600,_ cell concentration or CDW determination. It delivers detailed, advanced information about the growth conditions and expands the knowledge of limitations or crucial changes.

## Supplementary Material



## Figures and Tables

**Table 1.** Microorganisms and media used for sensor evaluation.

**Species**	**Strain**	**Medium**
*A. niger*	D15 Xyn2	“FM”
CHO	K1	TC-42 (TeutoCell) + 8 mM glutamine
*L. zeae*	DSM 20178	MRS
*K. pastoris*	DSM 70382	YM

**Table 2.** Cultivation parameters used for the sensor evaluation.

**Species**	**T. [°C]**	**Agitation [rpm]**	**Flask Type [mL]**	**Volume [mL]**	**Preculture [h]**
*A. niger*	37	150	500 WB	250	20
CHO-K1	37	130	500 NB	200	144
*L. zeae*	37	100	500 NB	100	48
*K. pastoris*	30	150	500 WB	100	18

WB = with baffles, NB = non baffled

**Table 3.** Calibration validation results in comparison of two ranges. CDW in g·L^−1^, cell concentration (CC) in 10^9^ cells·mL^−1^, CHO-K1 10^6^ cells·mL^−1^.

**Species**	**RMSEP**			**Error [%] Full Range**			**Error OD_600_ >a^1,2,^^3,^^4^ [%]**		

	**OD_600_**	**CDW**	**CC**	**OD_600_**	**CDW**	**CC**	**OD_600_**	**CDW**	**CC**

*L. zeae^1^*	0.18	0.15	0.39	5.54	11.12	9.33	3.33	11.12	3.75
*K. pastoris^2^*	0.39	0.12	0.10	9.39	12.58	7.11	5.35	7.32	4.29
*A. niger^3^*	–	0.37	–	–	7.23	–	–	6.65	–
CHO-K1^4^	–	–	0.76	–	–	13.05	–	–	7.91

1, 2 a = OD_600_ > 2

3 a = CDW >4.1 g·L^−1^

4 a = CC >7·× 10^5^ cells·mL^−1^

**Table 4.** Standard deviation of the offline measurement results. CDW in g·L^−1^, cell concentration (CC) in 10^9^ cells·mL^−1^, CHO-K1 10^6^ cells·mL^−1^.

**Species**	**Standard Deviation**			**Deviation / Measurement [%] Full Range**		
		
	**OD_600_**	**CDW**	**CC**	**OD_600_**	**CDW**	**CC**
*L. zeae*	0.06	–	–	1.5	–	–
*K. pastoris*	0.13	0.18^1^	0.18	1.3	3.2^1^	12.0

1 due to critical culture volume limitation no double determination could be carried out during cultivation, *S. cerevisiae* NCYC 1024 served as model for manual yeast CDW-determination, 10-fold determination with a mean biomass concentration of 5.5 g·L^−1^ was performed for error estimation.

## Abbreviations

CC: cell concentration
CDW: cell dry weight
CHO: Chinese hamster ovary
DLR: dual lifetime referencing
DMSO: dimethyl sulfoxide
DO: dissolved oxygen
NB: no baffles
NCYC: national collection of yeast cultures
NIR: near infrared
OD_x_: optical density at λ = x nm
PD: photodiode
RMSEP: root mean square error of prediction
SFR: shake flask reader
WB: with baffles
