# Supplementary material for: Application of an Online-Biomass Sensor in an Optical Multisensory Platform Prototype for Growth Monitoring of Biotechnical Relevant Microorganism and Cell Lines in Single-Use Shake Flasks
Source: Sensors (Basel). 2014 Sep 17;14(9):17390–405. doi: 10.3390/s140917390 (PMC4208230; doi:10.3390/s140917390)
Supplement: Supplementary file 1 [file sensors-14-17390-s001.pdf]

OPEN ACCESS
sensors
ISSN 1424-8220
www.mdpi.com/journal/sensors

Supplementary Information

Application of an Online-Biomass Sensor in an Optical Multisensory Platform Prototype for Growth Monitoring of Biotechnical Relevant Microorganism and Cell Lines in Single-Use Shake Flasks. *Sensors* 2014, *14*, 17390-17405

Christian Ude [1], Jörg Schmidt-Hager [1], Michael Findeis [2], Gernot Thomas John [2], Thomas Scheper [1] and Sascha Beutel [1,*]

[1] Leibniz University of Hanover, Institute of Technical Chemistry, Callinstr. 5, 30167 Hannover, Germany; E-Mails: ude@iftc.uni-hannover.de (C.U.); Schmidt-hager@iftc.uni-hannover.de (J.S.-H.), scheper@iftc.uni-hannover.de (T.S.)

[2] PreSens Precision Sensing GmbH, Josef-Engert-Str. 11, 93053 Regensburg, Germany; E-Mails: Michael.Findeis@presens.de (M.F.); G.John@presens.de (G.T.J.)

* Author to whom correspondence should be addressed; E-Mail: beutel@iftc.uni-hannover.de; Tel.: +49-0511-762-2868; Fax: +49-0511-762-3004.

---

1. Cell Size Determination

```
// P. pastoris
run("Convolve...", "text1=[1 1 1\n1 -8 1\n1 1 1\n]");
run("8-bit");
setThreshold(50, 255);
run("Convert to Mask");
run("Fill Holes");
run("Watershed");
run("Open");
run("Analyze Particles...", "size=20-300 circularity=0.50-1.00 show=[Count
Masks] include summarize");
run("Invert");
close();close();

// L. zeae
run("Convolve...", "text1=[1 1 1\n1 -8 1\n1 1 1\n]");
run("8-bit");
setThreshold(50, 255);
```

```
run("Convert to Mask");
run("Watershed");
run("Analyze Particles...", "size=5-100 circularity=0.0-1.00 show=[Count Masks] include summarize");
run("Invert");
close();close();
```

2. VBA

```
Option Explicit

Public Sub AutoKorrelation()

Call Clear

Dim nosampl As Boolean
nosampl = Menu.MultiPage1.Page1.CheckBox6.Value
If nosampl = True Then Call AutoKorrelation2

Dim k, i, n, m, l, s, t, u As Integer
Dim threshold1 As Double
Dim threshold2 As Double

Dim A As New Collection
Dim A2 As New Collection
Dim B As New Collection
Dim B2 As New Collection
Dim C As New Collection
Dim C2 As New Collection

Dim Range1 As Long
Dim Range2 As String
Dim Range2a As String
Dim Range2b As String

Range1 = CLng(Menu.MultiPage1.Page1.TextBox3.Value)
Range2a = Menu.MultiPage1.Page1.TextBox4.Value
Range2b = Menu.MultiPage1.Page1.TextBox5.Value

threshold1 = CDbl(Menu.MultiPage1.Page1.TextBox1.Value)
threshold2 = CDbl(Menu.MultiPage1.Page1.TextBox2.Value) / 60

Select Case Menu.MultiPage1.Page1.CheckBox4.Value
Case Is = True: u = 0
```

```
Case Is = False: u = 1
End Select

k = 2
l = 1
A.Add (0)
B.Add (Tabelle1.Cells(46, k + 2)) 'Amp bei t0
If Tabelle1.Cells(2, k) = 0 And Tabelle2.Cells(RCC1(Range2a), RCC2(Range2a)) = 0 Then C.Add Tabelle2.Cells(RCC1(Range2b), RCC2(Range2b))
For n = 2 To Range1

    If (Tabelle1.Cells(n + 1, k) - Tabelle1.Cells(n, k)) > threshold2 Then

        For s = RCC1(Range2a) To RCC3(Range2a)

            If (Tabelle1.Cells(n, k) - Tabelle2.Cells(s, RCC2(Range2a))) ^ 2 < threshold1 Then
                A.Add Tabelle1.Cells(n + u, k)
                B.Add Tabelle1.Cells(n + u, k + 2)
                C.Add Tabelle2.Cells(s, RCC2(Range2b))
                l = l + 1
            End If

        Next s

    End If

Next n


m = 0
i = 1
For i = 1 To l

    If i = l Then
    A2.Add (A(i))
    B2.Add (B(i))
    C2.Add (C(i))
    GoTo Ende1
    End If

    If A(i) <> A(i + 1) Then
    A2.Add (A(i))
```

```
    B2.Add (B(i))
    C2.Add (C(i))
    m = m + 1
    End If

Next i
Ende1:


t = 8
i = 1
For n = 2 To m + 2

    Tabelle1.Cells(n, t) = A2(i)
    Tabelle1.Cells(n, t + 1) = B2(i)
    Tabelle1.Cells(n, t + 2) = C2(i)

    i = i + 1

Next n


End Sub

Public Sub Clear()

Tabelle1.Range("H2:J400000") = ""

If Menu.TextBox2.Value < 0.3 Then
Menu.TextBox2.Value = 0.3
MsgBox "minimum value = 0.3", vbExclamation, "Threshold Error"
End If

End Sub
```

```
Option Explicit

Public Sub MedianKorrektur()

Application.ScreenUpdating = False

Call Clear2

Dim k, i, n, m, l, s As Integer
Dim Range1 As Long
Dim fenster As Integer
Dim threshold2 As Double
Dim dAMP As Double
Dim discdata As Boolean
Dim fwdmed As Boolean
Dim speedmodeon As Boolean

Range1 = CLng(Menu.MultiPage1.Page2.TextBox17.Value)
fenster = CInt(Menu.MultiPage1.Page2.TextBox6.Value)
threshold2 = CDbl(Menu.MultiPage1.Page1.TextBox2.Value) / 60
discdata = Menu.MultiPage1.Page2.CheckBox1.Value
fwdmed = Menu.MultiPage1.Page2.CheckBox2.Value
speedmodeon = Menu.MultiPage1.Page2.CheckBox3.Value

k = 3
l = 2

For n = fenster + 1 To Range1
    If Tabelle1.Cells(n - (fenster - 1), k - 2) <> "" Then
        Tabelle1.Cells(n - (fenster - 1), k - 1) = (Tabelle1.Cells(n - (fenster - 1), k - 2) - Tabelle1.Cells(2, 
k - 2)) / (3600000)
        Else: GoTo Ende1
    End If
Next n
Ende1:

For n = fenster + 1 To Range1

If Tabelle1.Cells(n, k) = "" Then GoTo Ende2

    If (Tabelle1.Cells(n, l) - Tabelle1.Cells(n - 1, l)) > threshold2 Then
```

```
        If discdata = False And fwdmed = False Then GoTo marke1:

            If discdata = True Then
                n = n + fenster
                GoTo marke1
            End If

            dAMP = Application.Median(Tabelle1.Range(Cells(n + 2 * (fenster - 1), k), Cells(n +
(fenster - 1), k))) -
           Application.Median(Tabelle1.Range(Cells(n + fenster, k), Cells(n + 1, k)))

                s = 0
                Do
                Tabelle1.Cells(n, k + 1) = Application.Median(Tabelle1.Range(Cells(n + (fenster - 1),
k), Cells(n, k))) - dAMP
                s = s + 1
                n = n + 1
                Loop While s < fenster

            n = n - 1

    End If

marke1:
    Tabelle1.Cells(n, k + 1) = Application.Median(Tabelle1.Range(Cells(n - (fenster - 1), k), Cells(n,
k)))

Next n
Ende2:


If speedmodeon = True Then
Call SpeedMode
Else: Call noSpeedModeAmp
End If

Application.ScreenUpdating = True

End Sub


Public Sub Clear2()
```

```
Dim discdata As Boolean
Dim fwdmed As Boolean
discdata = Menu.MultiPage1.Page2.CheckBox1.Value
fwdmed = Menu.MultiPage1.Page2.CheckBox2.Value

Tabelle1.Range("B2:B400000") = ""
Tabelle1.Range("D2:D400000") = ""

If discdata = True And fwdmed = True Then
discdata = False
fwdmed = False
MsgBox "Wählen Sie eine der beiden Methoden oder keine", vbExclamation, "method incompability"
End If

End Sub
```

© 2014 by the authors; licensee MDPI, Basel, Switzerland. This article is an open access article distributed under the terms and conditions of the Creative Commons Attribution license (http://creativecommons.org/licenses/by/3.0/).
